# Retrieving Sun-Induced Chlorophyll Fluorescence from Hyperspectral Data with TanSat Satellite

**DOI:** 10.3390/s21144886

**Published:** 2021-07-18

**Authors:** Shilei Li, Maofang Gao, Zhao-Liang Li

**Affiliations:** 1 Key Laboratory of Agricultural Remote Sensing, Ministry of Agriculture and Rural Affairs, Institute of Agricultural Resources and Regional Planning, Chinese Academy of Agricultural Sciences, Beijing 100081, China; 82101201641@caas.cn (S.L.); lizhaoliang@caas.cn (Z.-L.L.); 2 State Key Laboratory of Resources and Environmental Information System, Institute of Geographic Sciences and Natural Resources Research, Chinese Academy of Sciences, Beijing 100101, China

**Keywords:** singular value decomposition (SVD), solar-induced chlorophyll fluorescence (SIF), TanSat satellite, Bayesian Information Criterion (BIC)

## Abstract

A series of algorithms for satellite retrievals of sun-induced chlorophyll fluorescence (SIF) have been developed and applied to different sensors. However, research on SIF retrieval using hyperspectral data is performed in narrow spectral windows, assuming that SIF remains constant. In this paper, based on the singular vector decomposition (SVD) technique, we present an approach for retrieving SIF, which can be applied to remotely sensed data with ultra-high spectral resolution and in a broad spectral window without assuming that the SIF remains constant. The idea is to combine the first singular vector, the pivotal information of the non-fluorescence spectrum, with the low-frequency contribution of the atmosphere, plus a linear combination of the remaining singular vectors to express the non-fluorescence spectrum. Subject to instrument settings, the retrieval was performed within a spectral window of approximately 7 nm that contained only Fraunhofer lines. In our retrieval, hyperspectral data of the O_2_-A band from the first Chinese carbon dioxide observation satellite (TanSat) was used. The Bayesian Information Criterion (BIC) was introduced to self-adaptively determine the number of free parameters and reduce retrieval noise. SIF retrievals were compared with TanSat SIF and OCO-2 SIF. The results showed good consistency and rationality. A sensitivity analysis was also conducted to verify the performance of this approach. To summarize, the approach would provide more possibilities for retrieving SIF from hyperspectral data.

## 1. Introduction

The solar energy absorbed by vegetation is released in the form of optical signals, namely sun-induced chlorophyll fluorescence (SIF), which is closely related to photosynthesis [1,2]. The spectral emission of SIF spans from 650 nm to 800 nm, with two peaks located around 685 and 740 nm [3,4]. SIF can be used as a probe for photosynthesis [5,6,7]. Additionally, SIF is also used to study drought [8,9], vegetation stress [10,11], vegetation phenology [12,13] and crop yield prediction [14,15,16].

The first global maps of SIF were produced by Frankenberg et al. [17] and Joiner et al. [18], which also opened the door to large-scale monitoring of global vegetation productivity. Subsequently, more related research has been reported gradually. Guanter et al. [19] proposed an SIF retrieval approach based on singular vector decomposition (SVD) technology and retrieved global SIF using Japanese Greenhouse Gases Observing Satellite (GOSAT) data. Joiner et al. [20] utilized a physical model-based algorithm that focused on in-filling of Fraunhofer lines, to retrieve global SIF from GOSAT and the Scanning Imaging Absorption Spectrometer for Atmospheric Chartography (SCIAMACHY) data. Based on principal component analysis (PCA) technology, Joiner et al. [21] described a novel methodology to retrieve global far-red SIF using Global Ozone Monitoring Instrument 2 (GOME-2) data. Similarly, Frankenberg et al. [22] and Guanter et al. [23] evaluated the feasibility and potential of using the Orbiting Carbon Observatory-2 (OCO-2) and the TROPOspheric Monitoring Instrument (TROPOMI), respectively, to retrieve SIF. Furthermore, a series of SIF were retrieved [24,25,26,27,28] based on the above studies.

In the process of global SIF retrieval development, data-driven algorithms are widely used and have become mainstream because of their simple principles and convenient operations. Data-driven algorithms are composed of two categories: PCA-based and SVD-based retrieval approaches. Although they are similar, their practical applications are different. Currently, the PCA-based algorithms use relatively coarse spectral resolution (0.5 nm) data to retrieve SIF in broad spectral windows (>30 nm) [21,23,24]. The SVD-based algorithms retrieve SIF using ultra-high spectral resolution (<0.05 nm) data in narrow spectral windows (~2 nm), with the assumption that SIF remains constant within the spectral window while ignoring atmospheric effects [19,22,28]. There is no relevant report on the attempt of retrieving SIF using ultra-high spectral resolution remotely sensed data in a broad spectral window. Building upon the work of Guanter et al. [19], we described an SVD-based approach that is applicable to a broad spectral windows and does not assume a constant SIF for the retrieval of when using ultra-high spectral data. The performance of this approach was evaluated using the Chinese carbon dioxide observation satellite mission (TanSat).

## 2. Materials and Methods

### 2.1. Materials

#### 2.1.1. TanSat Satellite Data

TanSat was launched in December 2016. It is a sun-synchronous satellite that orbits at an altitude of approximately 700 km with an equatorial crossing time near 13:30 local solar time and a revisit period of 16 days. TanSat is equipped with two instruments: the high spectral resolution Atmospheric Carbon Dioxide Grating Spectroradiometer (ACGS) and the Cloud and Aerosol Polarimetry Imager (CAPI) that monitor CO_2_ and aerosols, respectively. The ACGS has three sets of grating spectrometers that support the detection of O_2_ and CO_2_ absorption spectra in three channels: the O_2_-A band at 760 nm (758–778 nm), a weak CO_2_ band at 1.6 μm (1594–1624 nm), and a strong CO_2_ band at 2.06 μm (2041–2081 nm). The spectral resolution of ACGS is 0.044 nm in the O_2_-A band, 0.12 nm in the weak CO_2_ band, and 0.16 nm in the strong CO_2_ band [28,29]. TanSat has three observation modes: nadir, sun-glint, and target modes. In this work, we used Level 1B data of the nadir mode from ACGS [30,31].

#### 2.1.2. SIF Products

To evaluate the reliability of the retrieval approach and minimize errors caused by instrument, we compared SIF retrievals with TanSat SIF. For further verification, OCO-2 SIF datasets were also used. Both the TanSat SIF and OCO-2 SIF datasets were retrieved using the spectral window of around 758 nm and 770 nm. Here, we selected SIF_758_ for comparison, because SIF_770_ is weakly affected by oxygen absorption [19]. TanSat SIF and OCO-2 SIF datasets are available online at http://data.casearth.cn/sdo/detail/5d905086088716491c0cc1f4 (accessed on 23 January 2021) and https://disc.gsfc.nasa.gov/datasets?keywords=OCO-2&page=1 (accessed on 26 February 2021), respectively.

### 2.2. Retrieval Methodology

#### 2.2.1. Fundamental Basis

Assuming that a fluorescent target observed by satellite sensor to be Lambertian, the radiance $L_{TOA}$ received by a satellite sensor could be described by:

(1) $$L_{TOA}=\frac{\rho_{P}}{\pi }\cdot I_{sc}\cdot \mu_{0}+F_{s}\cdot h_{F}\cdot T_{↑}$$

where $\rho_{P}$ is the planetary reflectance, $I_{sc}$ is the solar irradiance at the top of the atmosphere (TOA), $\mu_{0}$ is the cosine of the solar zenith angle, $F_{s}$ is the amount of SIF, $h_{F}$ is the normalized reference fluorescence emission spectrum, and $T_{↑}$ is the atmospheric transmittance from ground to sensor. Splitting $\rho_{P}$ into the contribution of the surface reflectance ($\rho_{s}$) and the atmospheric path reflectance ($\rho_{0}$), Equation (1) is formulated as

(2) $$L_{TOA}=\frac{I_{sc}\cdot \mu_{0}}{\pi }\cdot \left[\rho_{0}+\frac{\rho_{s}T_{↓↑}}{1-S\rho_{s}}\right]+\frac{F_{s}T_{↑}}{1-S\rho_{s}}$$

where S is the atmospheric spherical albedo and $T_{↓↑}$ is the sun-to-satellite (two-way) total atmospheric transmittance. Additionally, $\rho_{P}$ can be modeled by low and high-frequency components. The spectrally smooth low-frequency components ($\rho_{s}$, $\rho_{0}$, and S), can be presented by a polynomial of the order *n* in wavelength. High-frequency information can be regarded as a linear superposition of a small set of atmospheric principal components. Following Guanter et al. [23] and Köhler et al. [24], Equation (2) is further written as

(3) $$L_{TOA}=\left(\overset{n_{P}}{\underset{i=0}{\sum }}a_{i}\cdot \lambda^{i}\right)\cdot \left(\overset{n_{v}}{\underset{j=1}{\sum }}\omega_{j}\cdot v_{j}\right)+F_{s}\cdot h_{F}\cdot T_{↑}$$

where a is the coefficient of wavelength λ, ω is the weight of singular vector ν, $n_{P}$ is the order of λ, and $n_{v}$ is the number of singular vectors (SVs). Generally, the first singular vector carries the most important information and determines the shape of the spectrum. Additionally, the ground-to-sensor transmittance ($T_{↑}$) can be ignored if the retrieval window is free from atmospheric features. Based on Guanter et al. [19] and Guanter et al. [23], the final form of our model was expressed as

(4) $$L_{TOA}=v_{1}\cdot \overset{n_{P}}{\underset{i=0}{\sum }}a_{i}\cdot \lambda^{i}+\overset{n_{v}}{\underset{j=2}{\sum }}\omega_{j}\cdot v_{j}+F_{s}\cdot h_{F}$$

According to the findings in the study by Guanter et al. [32], the second-order polynomial is sufficient to describe the low-frequency contribution in the fitting window < 15 nm width. In view of the 7 nm spectral window in this study, we used the first-order polynomial ($n_{P}$ = 1) to express low-frequency contribution. To determine the optimal number of SVs, the Bayesian Information Criterion (BIC) is adopted to self-adaptively select $n_{v}$ [33]. The rule of using BIC is to calculate the corresponding BIC values when using different numbers of SVs to reconstruct a TOA spectrum. The optimal number of SVs can be set according to the corresponding minimum BIC value. The BIC is calculated as

(5) $$\mathrm{BIC}=n_{\lambda }\ln\left(RSS/n_{\lambda }\right)+k\ln\left(n_{\lambda }\right)$$

where $n_{\lambda }$ is the number of spectral channels, k is the number of coefficients, and RSS is the residual sum of squares between the modeled radiance and measured radiance. It is calculated as follows:

(6) $$RSS=\overset{n_{\lambda }}{\underset{\lambda =n_{1}}{\sum }}\varpi \left(\lambda \right){\left[L\left(\lambda \right)-\tilde{L}\left(\lambda \right)\right]}^{2}$$

where L is the measured total upwelling radiance, $\tilde{L}$ is the reconstructed radiance, ϖ is the reciprocal of the uncertainty of radiance, and it is formulated as follows:

(7) $$\varpi \left(\lambda \right)=\frac{1}{u_{n}\left(\lambda \right)}=\frac{SNR\left(\lambda \right)}{L\left(\lambda \right)}$$

where $u_{n}$ is the uncertainty due to sensor noise, and SNR refers to the signal-to-noise ratio of the instrument.

#### 2.2.2. Generation and Assessment of SVs

**Figure 1:**
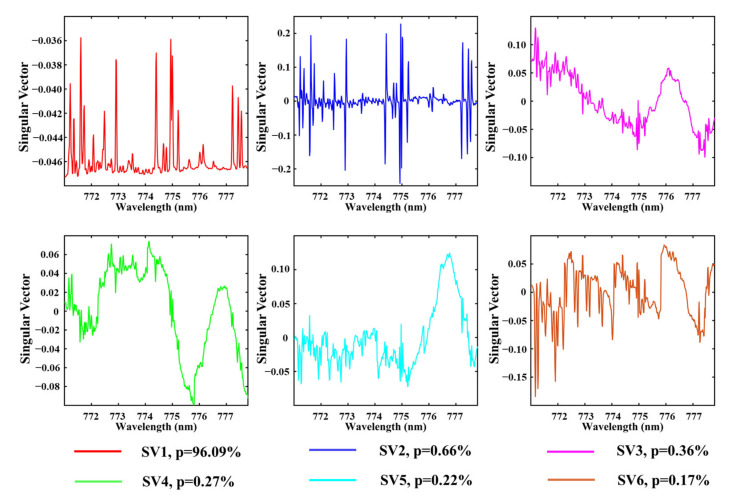
The first six SVs derived from the training set, p refers to the contribution of each ν to the total variance of the training set.

In our model, the selection of training spectra is critical. To select spectra that did not contain any information of vegetation while ensuring their representative nature, we selected a training set that included more than 7500 soil and water spectra across the globe. Singular value decomposition (SVD) technology was employed to generate SVs from the training set [19]. The first six SVs and the weight of each singular vector in the total variance are displayed in Figure 1.

#### 2.2.3. Performance Evaluation Method

In this study, the performance of retrieved SIF was assessed using the coefficient of determination (*R*^2^), bias, and root mean square error (*RMSE*). The definitions of these indexes are as follows:

(8) $$R^{2}={\left[\frac{\sum_{i=1}^{n}\left(x_{i}-\bar{x}\right)\cdot \left(y_{i}-\bar{y}\right)}{\sqrt{\sum_{i=1}^{n}{\left(x_{i}-\bar{x}\right)}^{2}}\cdot \sqrt{\sum_{i=1}^{n}{\left(y_{i}-\bar{y}\right)}^{2}}}\right]}^{2}$$

(9) $$bias=\frac{\sum_{i=1}^{n}\left(x_{i}-y_{i}\right)}{n}$$

(10) $$RMSE=\sqrt{\frac{\sum_{i=1}^{n}{\left(x_{i}-y_{i}\right)}^{2}}{n}}$$

where $x_{i}$ and $y_{i}$ are the retrieved SIF and SIF product from TanSat or OCO-2, respectively. $\bar{x}$ and $\bar{y}$ are the averaged values of the retrieved SIF and SIF product, respectively, and n refers to the total number of SIF retrievals.

## 3. Results and Discussion

### 3.1. Reconstruction of Measured Spectra

**Figure 2:**
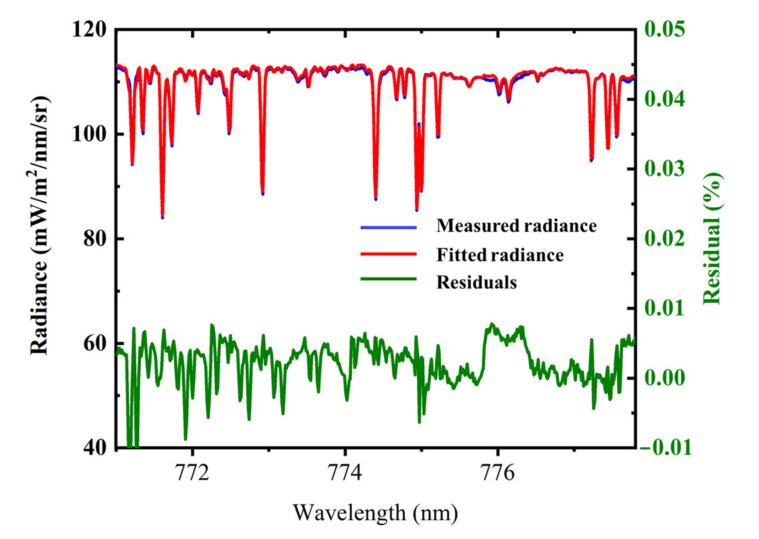
Exemplary spectral fit and its residuals in the spectral window of 771–778 nm. The left axis shows radiance, and the right axis corresponds to the residual.

Accurate input of model parameters is a prerequisite for SIF retrieval. BIC provides proper SVs to realistically restore the parameters in the model. Based on the final model given in Equation (4), we selected a spectral window of 771–778 nm that only contained several Fraunhofer lines to retrieve SIF. An example of reconstructed TOA radiance using the first five SVs (provided by BIC with the highest reconstruction accuracy) is depicted in Figure 2. Residuals in the model considering SIF are also plotted to illustrate that the measured radiance can be reconstructed with high accuracy.

### 3.2. SIF Retrievals

**Figure 3:**
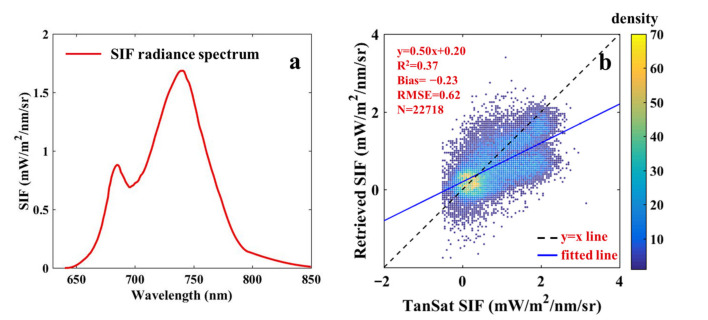
(**a**) SIF spectral distribution from SCOPE, (**b**) scatter plot of SIF retrievals and TanSat SIF. The color legend indicates the density of scattered points.

One-orbit hyperspectral measurements from the TanSat satellite were used to retrieve SIF. SIF retrievals at 775 nm were compared with TanSat SIF_758_ to evaluate the reliability of the retrieval approach. The Soil Canopy Observation, Photochemistry and Energy fluxes model (SCOPE) was also used to simulate a typical fluorescence spectrum to show the intuitive relationship of SIF at different bands (Figure 3a). SCOPE is an integrated model of radiation transmission and energy balance. It can simulate the spectra of TOC outgoing radiance, the reflectance factor, and fluorescence radiance for homogeneous canopies [33,34]. The comparison result is shown in Figure 3b. Although TanSat SIF was underestimated, it would not seriously affect the accuracy of SIF retrievals. According to the SIF spectral distribution, it showed a downward trend from 758 nm to 775 nm. Besides, the OCO-2 SIF product description mentions that SIF_758_ is ~1.5 times that of SIF_770_, and hence SIF_758_ should theoretically be about twice that of SIF_775_, which is confirmed by the slope in Figure 3b. The retrieved SIF in Figure 3b was obtained with the first five SVs and a standard deviation (σ) of 30 and was also regarded as a reference for the following sections.

**Figure 4:**
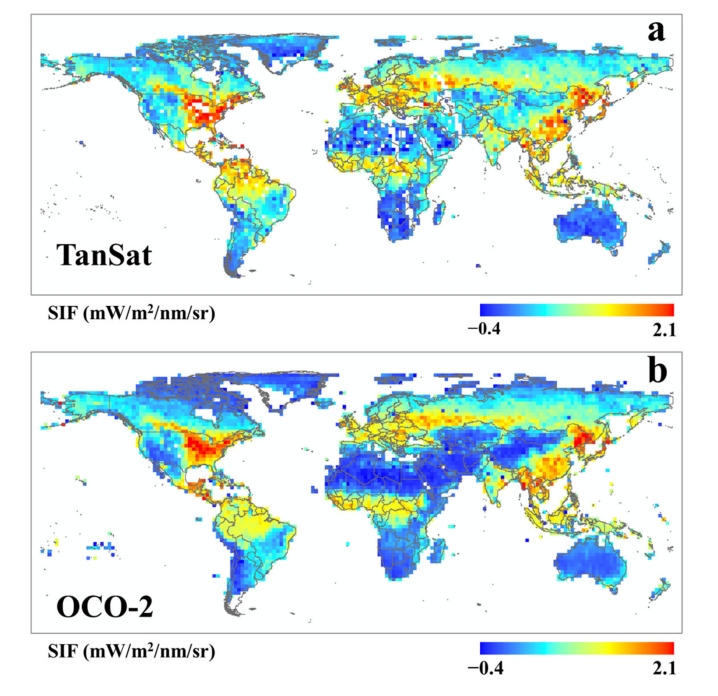
Global map of TanSat SIF_758_ (**a**) and OCO-2 SIF_758_ (**b**) in July 2017, with a cell resolution of 2° × 2°.

**Figure 5:**
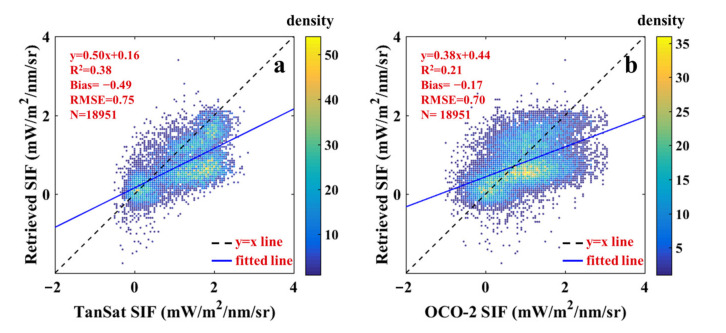
Scatter plots of SIF retrievals compared with TanSat SIF (**a**) and OCO-2 SIF (**b**). The color legend indicates the density of scattered points.

For further verification, we also made a comparison with OCO-2 SIF. Figure 4 shows the global distribution of TanSat SIF and OCO-2 SIF. Although there was good agreement on the whole, there were also local differences. As the footprints of different satellites did not completely correlate, the center distance that was between the verification points was controlled within 1 km. As shown in Figure 5, for the OCO-2 SIF, a similar underestimation was recognized, which proved the quality performance of this SIF retrieval approach. Additionally, the deterioration of the comparison results with that of OCO-2 SIF might be due to the performance of these two sensors, different algorithms, and mismatched footprints.

### 3.3. Sensitivity Analysis

The data-driven algorithm was semi-empirical, which means that the selection of parameters in the model would lead to uncertainty in the retrieval results. In this section, we conducted a sensitivity analysis of the retrieval approach.

#### 3.3.1. Width of the Fluorescence Emission Spectrum

**Figure 6:**
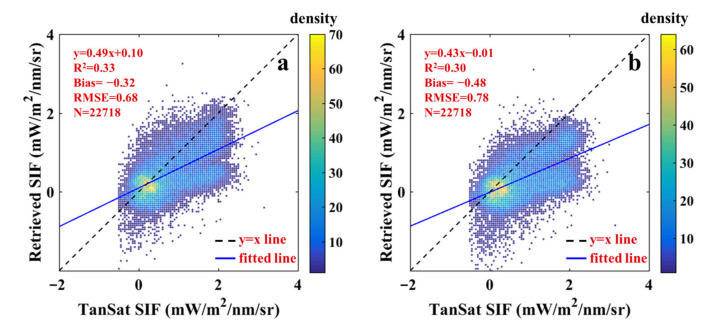

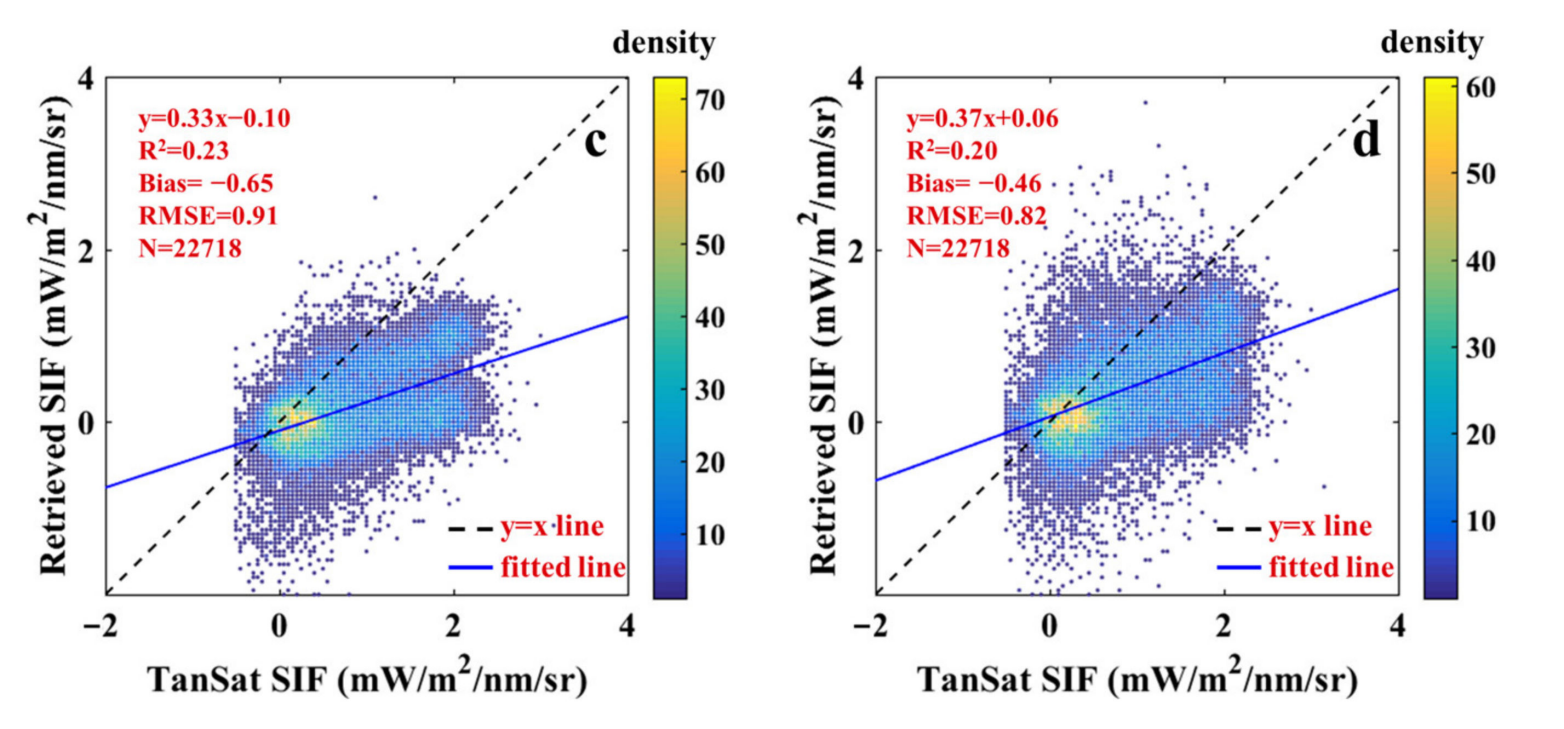
Scatter plot of SIF retrievals and TanSat SIF with different standard deviations: 20 (**a**), 25 (**b**), 35 (**c**), 40 (**d**). The color legend indicates the density of scattered points.

$h_{F}$, an important parameter, provides the shape of the fluorescence emission spectrum in the model. The spectral function is generally expressed in the form of a Gaussian function. It is formulated as

(11) $$h_{F}=\varphi e^{-\frac{{\left(\lambda -740\right)}^{2}}{2\sigma^{2}}}$$

where φ is the height of the function spike and defaults to 1, λ refers to the wavelength, and σ is the standard deviation that characterized the width of the fluorescence emission spectrum. In this study, we considered the standard deviation and explored the influence of spectral widths on the retrieval results. The SIF retrievals with different values of σ are plotted in Figure 6. Although it was generally underestimated, there were still some differences. When σ was larger than 30 (Figure 6c,d), the underestimation was more obvious than when it was less than 30, and the RMSE also increased significantly. Under these circumstances, TanSat SIF was more than twice the retrieval results, which demonstrated that the value of σ was unreasonable. The results in Figure 6a,b and Figure 3a show that when σ was 20 or 30, the relationship between the SIF retrievals and TanSat SIF was more reasonable. Based on R^2^ and RMSE values, we inferred σ value of 30 to be the best choice.

**Figure 7:**
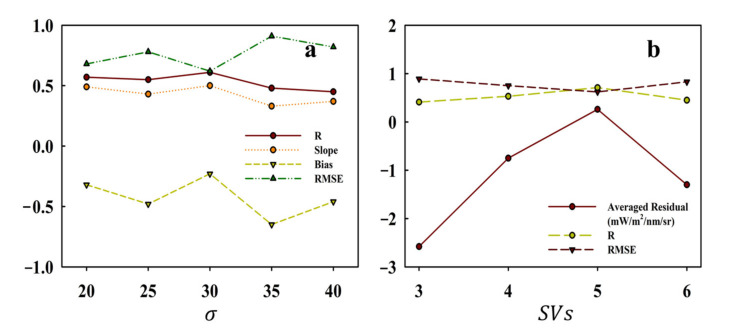
Sensitivity analysis results of different σ (**a**) and SVs (**b**).

The width of the fluorescence emission spectrum shaped the $h_{F}$ and also concerned SIF retrievals. For the conventional spectral window of 771–778 nm, $h_{F}$ was equivalent to providing a slope, and its similarity with the real fluorescence spectrum determined the retrieval error. The results of selecting different σ values were reproduced and are displayed in Figure 7a. It can be intuitively seen that when σ is 30, the correlation coefficient (R) and slope were the largest, while the bias and RMSE were the smallest. Wang et al. [35] explored the influence of σ on the accuracy of retrieval using simulated data, and found that the best retrievals (slope was close to 1 and bias was small) were obtained when σ was 30, which is consistent with the conclusion of this study. It is worth noting that the selection of σ would vary according to the distribution and length of different spectral windows because their positions in the fluorescence emission spectrum were different.

#### 3.3.2. Number of SVs

**Figure 8:**
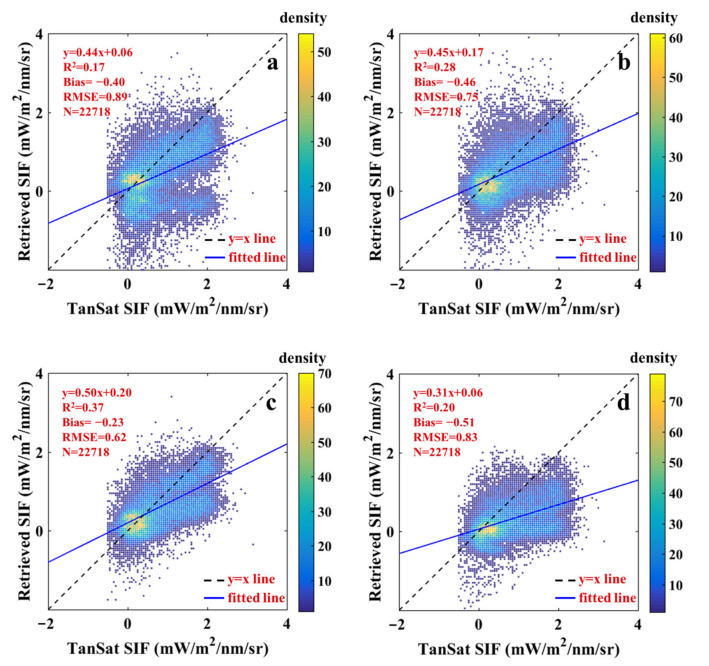
Scatter plot of SIF retrievals and TanSat SIF with different number of SVs: 3 (**a**), 4 (**b**), 5 (**c**), 6 (**d**). The color legend indicates the density of scattered points.

**Figure 9:**
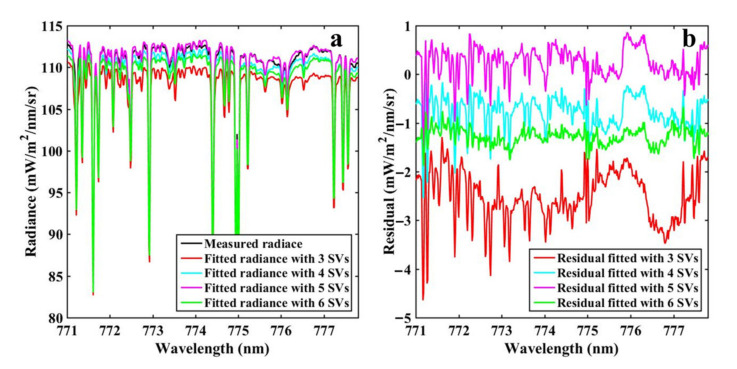
Spectral fits (**a**) and fit residuals (**b**) with different number of SVs.

The accuracy of spectral reconstruction determined the reliability of the retrieval results. Using SVs to characterize non-fluorescence spectrum was the driving basis for spectral reconstruction. Therefore, it was crucial to select an appropriate number of SVs in spectral reconstruction. Based on the BIC, we selected the first five SVs to model the original spectrum. To explore the influence of the number of SVs on the retrieval approach, we selected the first 3, 4, and 6 SVs to retrieve SIF and compared the retrievals with TanSat SIF. According to the results shown in Figure 8, increasing or decreasing the number of SVs reduced the correlation between SIF retrievals and TanSat SIF. The reconstruction accuracy of the spectrum was affected resulting in inaccurate retrievals, and the evidence is presented in Figure 9. The reconstructed spectra of the first five SVs were the most consistent with the measured spectra, and the corresponding residuals were also the smallest.

The selection of SVs depended on the spectral resolution and length of the spectral window. Usually, at a relatively low spectral resolution, a broader window requires more SVs to provide adequate fluorescence information for sufficient retrieval accuracy. The results of Wang et al. [35] confirmed this conclusion. Moreover, Köhler et al. [24] performed a similar experiment, and the results showed that as the window grows, the number of principal components (PCs) required also increases. Joiner et al. [25] stated that when the number of PCs in a fixed spectral window increases, the retrieval results will gradually stabilize, which indirectly validates this conclusion. Regarding the appropriate number of SVs, BIC can provide an ideal reference as previous studies have demonstrated that BIC is a reliable solution [24,33]. In this study, BIC was employed to determine the number of SVs to be used. As shown in Figure 7b, the first five SVs guaranteed high accuracy of spectral reconstruction, while having low RMSE and averaged residual.

#### 3.3.3. Selection of Spectral Window

**Figure 10:**
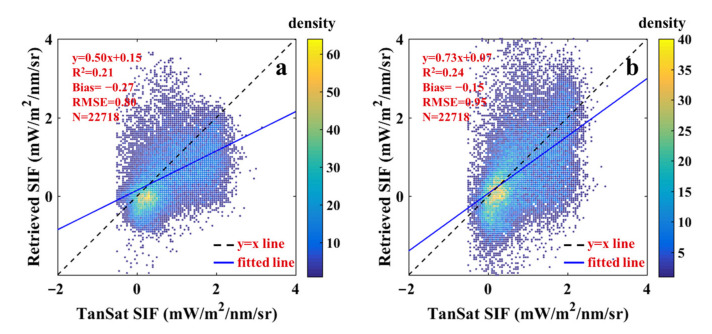
Scatter plot of SIF retrievals and TanSat SIF with different spectral windows: 769.5–776 nm (**a**) and 769.5–778 nm (**b**). The color legend indicates the density of scattered points.

The spectral window was also an important driving factor that determines the accuracy of SIF retrievals. Usually, the length of the spectral window and the spectral resolution determined the amount of SIF information provided. Owing to the limitations of the instrument, two additional spectral windows adjacent to the original window in Section 3.1, 769.5–776 nm and 769.5–778 nm, were selected to investigate the dependence of the retrieval results on the spectral window. Figure 10 depicts the performance of these two windows. Compared with Figure 3b, these retrieval results were consistent overall, however, they were more scattered, and R^2^ value dropped by more than 0.1. This might be caused by the small number of atmospheric features contained in the additional spectral window. Similar conclusions have also been found in Guanter et al. [32] and Joiner et al. [21] stating that the inclusion of an O_2_ band in the spectral window will reduce the accuracy of the retrieval results. This might be explained by the lack or inaccurate estimation of atmospheric transmittance. Even though the comparison was relatively poor, we can still see from Figure 10 that the distribution of the retrieval results was reasonable. This illustrated the feasibility of our retrieval approach.

### 3.4. The Potential of This Study

SVD-based algorithms are at present being applied in a narrow window while assuming that the SIF remains constant in the spectral window and ignores the influence of atmospheric absorption. In this paper, the approach we presented has the potential to improve the situation. It allows us to retrieve SIF using spectral window with ultra-high spectral resolution data that does not contain atmospheric features. Moreover, even if O_2_ or water vapor characteristics are included in the spectral window, we can retrieve SIF from hyperspectral data using this approach, provided that the atmospheric transmittance is correctly estimated. For the estimating transmittance, the methods of Guanter et al. [23] and Köhler et al. [26] will be helpful. The merit of our approach is that it does not require the assumption that the SIF remains constant and can operate in a broad spectral window.

## 4. Conclusions

This study proposed an approach for the retrieval of SIF from ultra-high spectral satellite data that can be applied in a broad spectral window without setting the SIF remains constant. The basic idea of retrieving SIF from space is that the high-frequency contribution of the atmosphere is mainly derived from the first singular vector, and the low-frequency contribution is provided by the n-order polynomial (first-order is adopted in this study). The retrieval approach was tested using spectra acquired by the TanSat satellite. SIF retrievals showed good agreement with TanSat SIF and OCO-2 SIF, which indicates the reliability of our approach.

In addition, a comprehensive sensitivity analysis was carried out from the perspective of the width of the fluorescence emission spectrum, number of SVs, and selection of the spectral window to demonstrate the effectiveness of our approach. We also proved that BIC can provide suitable SVs to achieve high-precision SIF retrieval. The retrieval approach presented in this paper provides more choices for SIF retrieval from space. This will help to better understand information on the functional status of vegetation from a global perspective.

## Data Availability

TanSat satellite data can be downloaded by National Satellite Meteorological Center (NSMC), http://satellite.nsmc.org.cn/portalsite/Data/DataView.aspx?SatelliteType=2&SatelliteCode=TANSAT# (accessed on 12 July 2018). TanSat SIF and OCO-2 SIF products are freely available from http://data.casearth.cn/sdo/detail/5d905086088716491c0cc1f4 and https://disc.gsfc.nasa.gov/datasets?keywords=OCO-2&page=1, respectively.
